# Rapunzel Syndrome: Clinical, Diagnostic and Forensic Aspects in Related Deaths—A Review of the Literature

**DOI:** 10.3390/jcm13237464

**Published:** 2024-12-08

**Authors:** Matteo Antonio Sacco, Saverio Gualtieri, Aurora Princi, Lucia Tarda, Alessandro Pasquale Tarallo, Luca Calanna, Stefano Lombardo, Jasmine Calafiore, Santo Gratteri, Isabella Aquila

**Affiliations:** Department of Medical and Surgical Sciences, Institute of Legal Medicine, University “Magna Graecia” of Catanzaro, Viale Europa, Loc. Germaneto, 88100 Catanzaro, Italy; matteoantoniosacco@gmail.com (M.A.S.); saverio.gualtieri@studenti.unicz.it (S.G.); aurora.princi@studenti.unicz.it (A.P.); lucia.tarda@studenti.unicz.it (L.T.); alessandropasquale.tarallo@studenti.unicz.it (A.P.T.); luca.calanna@studenti.unicz.it (L.C.); stefano.lombardo@studenti.unicz.it (S.L.); jasmine.calafiore@studenti.unicz.it (J.C.); gratteri@unicz.it (S.G.)

**Keywords:** Rapunzel syndrome, trichotillomania, trichophagia, psychiatry

## Abstract

**Background:** Rapunzel syndrome is a rare and severe form of trichobezoar, characterized by the presence of hair masses in the stomach that often extend into the bowel, resembling the legendary “Rapunzel’s” long hair. **Methods**: This review examines the clinical, diagnostic, forensic, and post-mortem aspects associated with Rapunzel syndrome, with a focus on cases resulting in mortality or those at high risk of death due to complications. In particular, the review systematically analyzes the existing literature on fatal cases of Rapunzel syndrome, emphasizing insights into risk factors, clinical manifestations, diagnostic methods, autopsy findings, and preventive measures to provide a focused understanding of these critical aspects. **Results:** The syndrome predominantly affects young females with a history of trichotillomania (hair-pulling) and trichophagia (hair-eating), often associated with underlying psychiatric conditions. Clinically, Rapunzel syndrome presents with non-specific gastrointestinal symptoms, including abdominal pain, vomiting, and malnutrition, which may complicate timely diagnosis. Diagnosis typically involves imaging techniques such as ultrasound, CT scans, and endoscopy, but cases often go unrecognized until complications like intestinal obstruction, perforation, or even fatal outcomes occur. Forensically, Rapunzel syndrome presents unique challenges, as misdiagnosis or delayed intervention can lead to fatalities that may raise questions in medico-legal investigations. Post-mortem investigations, particularly autopsies, have proven instrumental in elucidating rare complications and advancing understanding of the syndrome’s long-term effects. **Conclusions:** Increased awareness, timely diagnosis, and comprehensive evaluation, including autopsy studies, are essential to improve patient outcomes and reduce the potential for life-threatening complications in this rare yet serious condition.

## 1. Background

Rapunzel syndrome is a rare medical condition characterized by the presence of a trichobezoar, a mass of ingested hair that accumulates in the stomach and can extend into the small intestine [1,2,3,4,5,6,7,8,9,10,11,12,13,14]. The name comes from the fairy tale of Rapunzel, referring to the “tail” of the trichobezoar resembling her long hair.

In some cases, the condition can lead to fatal outcomes, especially when complications like bowel perforation or aspiration occur, often exacerbated by delayed recognition of the syndrome. The rarity of Rapunzel syndrome, coupled with its potential for sudden and severe clinical deterioration, highlights the need to better understand its pathophysiology and diagnostic challenges [14,15,16,17,18]. Therefore, to provide a clearer understanding of the risk factors, clinical manifestations, and causes of death associated with Rapunzel syndrome, we performed a systematic review of the literature, with a particular focus on cases involving death or emergency situations associated with a high risk of fatal complications.

This review aims to synthesize existing case reports and studies, emphasizing fatal cases and those at risk of fatal outcomes, to provide insights into the diagnostic processes, the role of autopsy investigations, and preventive measures to enhance early detection and reduce mortality. The aim of the study is to provide a comprehensive evaluation of the literature on Rapunzel syndrome. Particular emphasis is placed on analyzing causes of death in fatal cases, as well as on the pivotal role of autopsy investigations in uncovering the pathophysiological mechanisms underlying these outcomes. By deepening the state of the art on this rare and under-recognized condition, the study aims to improve clinical awareness and inform forensic, diagnostic, and therapeutic practices, ultimately contributing to enhanced management and prevention of life-threatening complications.

## 2. Materials and Methods

A systematic review of the literature was conducted using the scientific search engines PubMed and NCBI, focusing on studies related to Rapunzel syndrome, its clinical manifestations, diagnostic techniques, and outcomes, particularly in fatal cases or those at high risk of death. The following keywords and search terms were employed: “Rapunzel syndrome and emergency OR Rapunzel syndrome and death”, ensuring a broad and comprehensive capture of studies detailing the syndrome’s clinical, forensic, and autopsy findings.

The initial step involved screening the titles of the identified studies to assess their relevance to the research objectives. An initial systematic search of the PubMed database yielded 32 studies that matched the broad search criteria. Subsequently, abstracts of the papers were carefully reviewed to ensure they met the inclusion criteria, which included studies on the clinical presentation, diagnostic methods, surgical interventions, and autopsy findings associated with Rapunzel syndrome, as well as the forensic aspects of deaths related to the syndrome. After screening the titles and abstracts, 17 studies were selected for full-text review. Special attention was paid to those studies that reported cases of fatal outcomes, with particular focus on autopsy findings and forensic investigations, in order to gain insights into the causes of death and the role of post-mortem examinations in diagnosing this rare condition.

**Figure 1:**
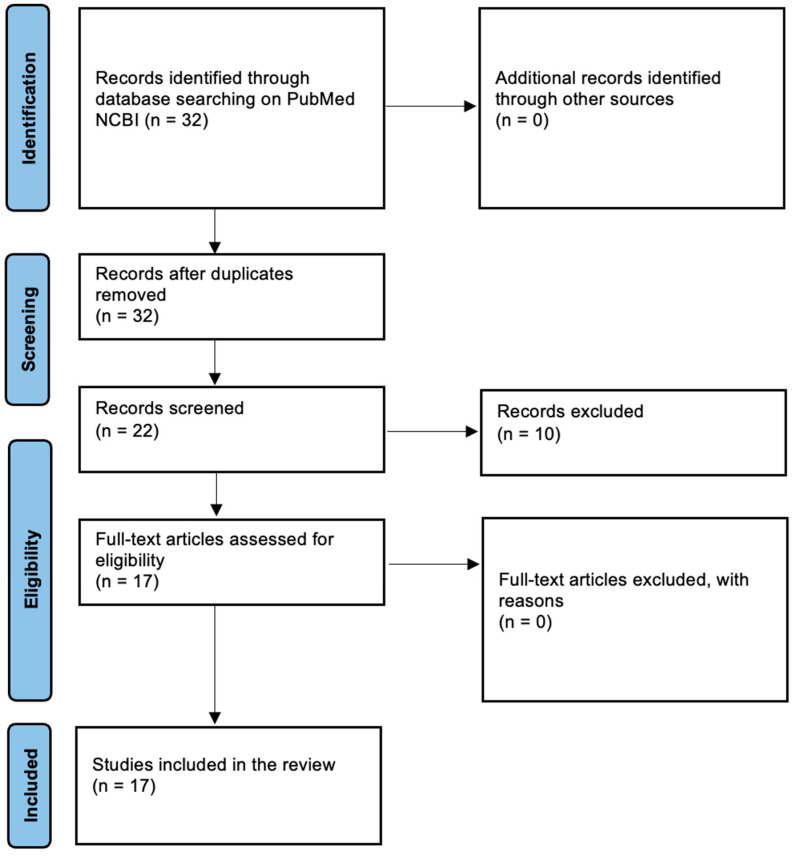
PRISMA flowchart.

The selected studies were a mix of case reports and retrospective analyses, with a significant focus on detailing autopsy findings and forensic investigations related to fatal cases of Rapunzel syndrome. The results from these studies were synthesized to identify patterns in clinical presentation, autopsy findings in cases of death, and bezoar dimensions (Figure 1).

## 3. Results

Most of these papers were case reports, while one was a retrospective study [19]. All the patients mentioned in these papers were females. Only three of these studies reported cases involving fatalities where autopsies were performed. In all other studies, the patients underwent surgery to remove the bezoar. Most of the patients were young: four were under 10 years old, one was 35 years old, and one was 55 years old, while the remainder were aged between 14 and 25 years. Liang et al. reported a retrospective study conducted on 10 patients with a median age of 9.1 years.

The most frequent clinical manifestations were abdominal pain (16 cases), vomiting (9 cases), anemia (5 cases), anorexia (4 cases), weight loss (4 cases), constipation (3 cases), nausea (2 cases), and fatigue (2 cases). Where information was available, bezoar dimensions varied significantly. In cases where patients died, autopsies were performed to determine the cause of death. In two cases, the cause of death was identified as bronchial aspiration of gastric material, caused by obstruction of the digestive tract [2,14]. In the third case, death resulted from peritonitis due to intestinal perforation [8]. In all three cases, the most indicative autopsy finding for diagnosing Rapunzel syndrome was the presence of large bezoar masses in the stomach and duodenum. In these cases, one patient was 10 years old, while the other two were less than 5 years old (Table 1).

## 4. Discussion

### 4.1. Clinical Aspects of Rapunzel Syndrome

#### 4.1.1. Causes of Rapunzel Syndrome

Rapunzel syndrome is intricately linked to psychological factors, particularly trichotillomania and trichophagia. Trichotillomania refers to the compulsive urge to pull out one’s hair, while trichophagia involves the subsequent ingestion of this hair, leading to the formation of trichobezoars in the gastrointestinal tract [1]. These psychological conditions are recognized as significant contributors to the development of Rapunzel syndrome, which is characterized by the migration of gastric trichobezoars into the small intestine, causing various digestive symptoms [2]. Genetic predispositions and family history also play roles in the development of Rapunzel syndrome. While the exact genetic mechanisms are not fully understood, there is evidence to suggest a hereditary component in trichotillomania and trichophagia, which can lead to the formation of trichobezoars [2]. Socio-environmental influences and stressors significantly impact the onset of Rapunzel syndrome. Stressful life events, trauma, and environmental factors can trigger or exacerbate compulsive behaviors like trichotillomania and trichophagia, ultimately leading to the formation of trichobezoars [3,4].

#### 4.1.2. Epidemiology of Rapunzel Syndrome

The incidence and prevalence rates of Rapunzel syndrome highlight its rarity across different populations. This syndrome is most frequently documented in medical literature as isolated cases or small series, emphasizing its uncommon occurrence [5]. While comprehensive epidemiological data are scarce, the few reports available suggest that the condition is significantly less prevalent than other gastrointestinal obstructions. The rarity of Rapunzel syndrome is further compounded by its association with specific psychiatric conditions, which may not be uniformly distributed across various demographics. This makes it challenging to determine precise prevalence rates, and thus, the syndrome remains a clinical curiosity with sporadic appearances in medical practice [1].

Age and gender play critical roles in the demographics commonly affected by Rapunzel syndrome. The condition predominantly occurs in adolescents and young females, with a notable gender bias favoring females [4]. This demographic trend can be attributed to the higher incidence of trichotillomania and trichophagia among young women, which are the primary psychiatric disorders linked to the formation of trichobezoars [3]. The psychological underpinnings, including stress and anxiety, may further influence these age and gender patterns, contributing to the development of such compulsive behaviors.

#### 4.1.3. Geographical Distribution of Rapunzel Syndrome

Rapunzel syndrome appears to have a higher incidence in specific regions, primarily due to differences in cultural practices and psychiatric prevalence. Certain areas report more cases, particularly where trichotillomania and trichophagia are more commonly diagnosed or culturally acknowledged [5]. These regions often have healthcare systems that are more adept at recognizing and reporting mental health disorders, leading to a higher identification rate of Rapunzel syndrome. Cultural factors play a significant role in the occurrence of Rapunzel syndrome. In societies where there is a higher stigma associated with mental health disorders, individuals might be less likely to seek treatment for conditions like trichotillomania and trichophagia, which are closely linked to the development of Rapunzel syndrome [5]. Cultural practices such as specific beauty standards that promote hair manipulation can also influence the syndrome’s occurrence. In some cultures, the pressure to maintain certain hairstyles may lead to increased hair pulling, subsequently increasing the risk of trichophagia. Additionally, cultural acceptance of unusual dietary practices can indirectly affect the syndrome’s incidence.

Differences in reporting and diagnosis of Rapunzel syndrome across countries are notable and can be attributed to disparities in medical knowledge and diagnostic capabilities. Some countries may have more advanced healthcare systems, which allow for better identification and documentation of rare conditions like Rapunzel syndrome [6]. Conversely, in regions where healthcare resources are limited, such syndromes might go undiagnosed or misdiagnosed, leading to underreporting.

#### 4.1.4. Clinical Manifestations of Rapunzel Syndrome

Rapunzel syndrome presents with a range of gastrointestinal symptoms that can have serious implications for the patient. Individuals often experience abdominal pain and discomfort due to the presence of a large trichobezoar obstructing the gastrointestinal tract, which can sometimes extend from the stomach into the intestines, causing severe complications such as bowel obstruction or perforation [4]. This obstruction can lead to nausea, vomiting, and early satiety, as the stomach’s capacity to process food is compromised. Furthermore, patients may suffer from unexplained weight loss and anemia, as the body’s ability to absorb nutrients is impaired [4].

Apart from the physical symptoms, Rapunzel syndrome is closely linked with behavioral indicators and psychiatric symptoms, such as anxiety, depression, or obsessive compulsive disorder, which require comprehensive psychiatric evaluation for effective management [6]. Patients may exhibit signs of emotional distress, such as social withdrawal and low self-esteem, which further complicate their condition by affecting their willingness to seek medical help.

### 4.2. Diagnostics and Prognosis of Rapunzel Syndrome

#### 4.2.1. Diagnosis of Rapunzel Syndrome

During medical examinations, several physical signs indicative of Rapunzel syndrome may be observed. Palpation of the abdomen might reveal a palpable mass, suggesting the presence of a trichobezoar [2]. Additionally, patients may present with signs of malnutrition or deficiencies, as the trichobezoar can interfere with nutrient absorption. There might also be visible signs of hair loss or bald patches on the scalp, resulting from trichotillomania, which can serve as a vital clue in diagnosing the syndrome [5].

The clinical criteria for identifying Rapunzel syndrome are crucial in distinguishing this rare condition from other gastrointestinal disorders. Conditions such as gastrointestinal tumors, chronic gastritis, and other forms of bezoars must be considered and excluded through comprehensive diagnostic evaluations [2]. To accurately diagnose Rapunzel syndrome, healthcare providers must recognize these signs and symptoms and consider them alongside the patient’s psychiatric history. The combination of psychiatric evaluation and physical symptomatology forms the foundation of the clinical criteria necessary for the diagnosis.

Early and accurate imaging is essential for guiding the appropriate surgical or medical intervention needed to manage Rapunzel syndrome effectively. Additionally, endoscopy may be utilized to visually confirm the presence of a trichobezoar and differentiate it from other potential masses or obstructions within the gastrointestinal tract.

#### 4.2.2. Prognosis of Rapunzel Syndrome

Recovery and recurrence rates in Rapunzel syndrome are influenced by several factors. Primarily, the underlying psychiatric condition plays a significant role, as individuals with persistent trichotillomania and trichophagia are more prone to recurrence [3]. The effectiveness of psychiatric treatment and therapeutic interventions greatly affects the likelihood of recovery. Additionally, early diagnosis and treatment can lead to more favorable outcomes, reducing the chances of recurrence.

In particular:

- The presence of other psychiatric comorbidities can complicate the treatment process.
- The patient’s age and overall health condition also contribute to recovery rates.
- Compliance with medical advice is crucial for preventing recurrence.

Without appropriate intervention, the syndrome can lead to severe complications, including intestinal obstruction, perforation, and even death [16]. On the other hand, patients who receive timely surgical removal of the trichobezoar combined with psychiatric support often experience improved outcomes.

#### 4.2.3. Pathophysiology and Mechanisms

Understanding the formation of trichobezoars is crucial to comprehending Rapunzel syndrome [16]. The formation process begins when an individual ingests hair, which is indigestible and accumulates over time in the gastrointestinal tract. This accumulation forms a dense mass called a trichobezoar [2]. The continuous ingestion of hair leads to the gradual growth of the trichobezoar, which eventually becomes large enough to cause significant gastrointestinal complications. In individuals with Rapunzel syndrome, the large trichobezoar can obstruct the gastrointestinal tract, leading to a range of symptoms such as abdominal pain, nausea, vomiting, and early satiety [7]. These symptoms occur due to the physical blockage created by the hairball, which prevents the normal passage of food and digestive fluids through the digestive system. Furthermore, the presence of a trichobezoar can lead to more severe outcomes like gastric or intestinal perforation, which can be life-threatening if not promptly addressed [14].

#### 4.2.4. Rapunzel Syndrome-Related Deaths

In contrast to living subjects, the analysis of documented cases in deceased subjects underscores the severity of Rapunzel syndrome when left untreated or undiagnosed. The documented fatalities were predominantly female, with the majority occurring in young children, pointing to a need for heightened awareness and monitoring in pediatric healthcare settings. Early intervention could potentially prevent such outcomes, underscoring the necessity for prompt medical attention when symptoms of trichophagia are observed. Treatment efficacy is notably higher when a multidisciplinary approach is employed, addressing both the physical removal of the trichobezoar and the underlying psychological issues. On the other hand, the few fatal cases underline the critical nature of early diagnosis and comprehensive treatment plans. Key factors contributing to successful outcomes include early detection, appropriate surgical techniques, and ongoing psychiatric support to prevent recurrence [30,31,32,33,34,35,36,37,38,39,40,41,42,43,44,45,46,47,48].

Cases of mortality linked to Rapunzel syndrome often arise from severe complications that are exacerbated by delayed diagnosis [1]. The obstruction can lead to severe gastrointestinal distress, including perforation, peritonitis, and even sepsis if not treated promptly. The risk of mortality increases significantly when trichobezoars cause these serious complications, as they can severely disrupt the body’s normal functions [6]. However, in deceased subjects, large trichobezoars can sometimes cause sudden death without prior symptomatology, making the condition particularly insidious [2].

From the literature analysis, it is evident that data on deaths of patients with Rapunzel syndrome are very limited. In this review, through the keywords, we evidenced only three deaths. In the other cases, patients underwent emergency surgery for the mass removal. Where death occurred, the patients were young (especially <5 years) and death occurred due to bronchoaspiration of gastric material. In particular, Matějů. E. et al. [14] reported the case of a 3-year-old girl whose death caused by bronchoaspiration of gastric material was associated with poor hygiene and care of the child by the parents. Zyla et al. [8] demonstrated the importance of using postmortem CT and MRI to identify trichobezoars. Liang et al. [19] performed a retrospective study analyzing the clinical history of 10 females (median age 9.1 years) who presented with abdominal pain, nausea, and vomiting. Complications occurred in six cases like small bowel obstruction and intestinal perforation, and all patients underwent surgical treatment.

#### 4.2.5. The Role of Autopsy in Rapunzel Syndrome Deaths

**Figure 2:**
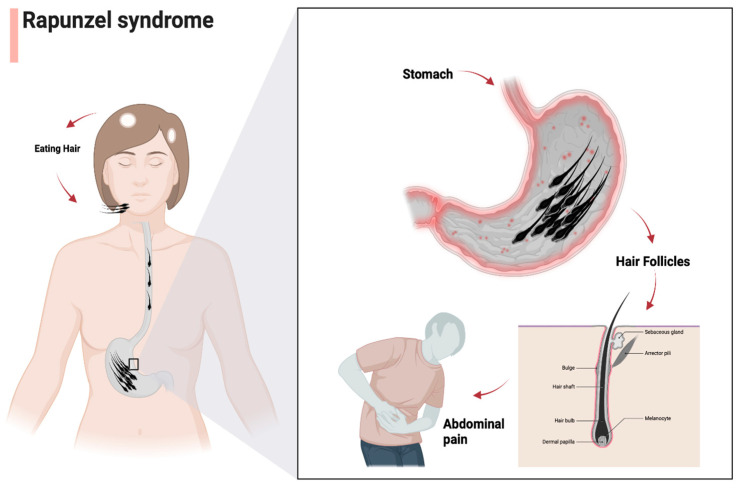
Pathological mechanisms of Rapunzel syndrome.

Establishing the cause of death in suspected Rapunzel syndrome cases is a critical aspect of forensic pathology, particularly given the unusual nature of the condition (Figure 2), which can lead to severe complications and even death, especially in young children [2]. Autopsies play a vital role in confirming the presence of trichobezoars and identifying any associated complications that may have contributed to the fatal outcome [5].

A comprehensive autopsy involves a meticulous examination of the gastrointestinal tract to identify and retrieve the trichobezoar [4]. Such detailed examinations can reveal the size and extent of the trichobezoar and any resulting damage to the stomach or intestinal walls, such as perforations or ulcerations. Additionally, autopsy can uncover other potential contributing factors, such as nutritional deficiencies or infections that may have exacerbated the patient’s condition. In a documented case involving a 4-year-old girl, autopsy findings were crucial in understanding the rapid progression of symptoms leading to her untimely death, which occurred shortly after a meal and a series of vomiting episodes [2]. These findings underscore the necessity of thorough autopsy protocols to ensure accurate identification of Rapunzel syndrome and its complications, which is essential for both medical and familial closure.

The legal and medical implications of autopsy findings in Rapunzel syndrome are significant, influencing both the understanding of the condition and the management of similar cases in the future. From a legal perspective, autopsy results can clarify the circumstances surrounding a death, potentially ruling out external factors such as foul play or neglect. This is particularly relevant in pediatric cases, where the sudden death of a child can lead to intense scrutiny and legal investigation [2]. Medically, the insights gained from autopsy findings contribute to the broader knowledge base of Rapunzel syndrome, informing the development of diagnostic criteria and treatment protocols. For instance, understanding the rapid onset of symptoms and the potential for asymptomatic periods, as revealed through autopsy, can guide clinicians in identifying at-risk patients and implementing preventive measures [2].

#### 4.2.6. Internal Signs at Autopsy in Rapunzel Syndrome Deaths

At autopsy, one of the most significant internal signs observed in Rapunzel syndrome deaths is the presence of trichobezoars in the gastrointestinal tract. These hairballs, formed by ingested hair, can be massive and extend from the stomach into the small intestine, a hallmark feature of Rapunzel syndrome [1]. The trichobezoars can remain undetected for extended periods, often not causing noticeable symptoms until they reach a size that can rapidly lead to severe complications [2]. This hidden progression makes the trichobezoars particularly dangerous, as they can lead to sudden death without prior warning [5].

Another critical aspect to examine during autopsy is the impact of trichobezoars on internal organs and systems. The presence of a large trichobezoar can exert considerable pressure on surrounding organs, affecting their function. This pressure can lead to complications such as gastric ulcers, perforation, or even pancreatitis due to the obstruction of the pancreatic duct. Additionally, the weight and size of the trichobezoar may cause the stomach and intestines to distend significantly, which can impede blood flow and result in ischemia. These changes can manifest as severe abdominal pain and other systemic symptoms, though they might not be evident until the condition becomes critical [2]. Evidence of intestinal obstruction or perforation is another significant internal sign observed at autopsy in Rapunzel syndrome cases [8].

From a forensic perspective, it is recommended that autopsy protocols in such cases include a complete examination of the gastrointestinal tract, extending from the esophagus to the rectal ampulla. This thorough approach facilitates the identification of hair residues or accumulations that may impact the intestines or surrounding organs.

#### 4.2.7. External Signs at Autopsy in Rapunzel Syndrome Deaths

During an autopsy of individuals suspected to have died from Rapunzel syndrome, a meticulous physical examination is crucial for identifying signs of malnutrition or dehydration. These symptoms are often prevalent due to the prolonged presence of hairballs in the gastrointestinal tract, which can obstruct the normal absorption of nutrients and fluids. Autopsy findings may reveal a significant decrease in body fat and muscle mass, which are indicative of chronic malnutrition. Additionally, the skin may appear dry and inelastic, further suggesting dehydration [6]. Another critical aspect of the external examination during an autopsy in Rapunzel syndrome cases is the identification of hair loss or bald patches, which may indicate trichotillomania. Trichotillomania, a compulsive behavior characterized by the urge to pull out one’s hair, often leads to visible bald spots on the scalp. This behavior is closely associated with the ingestion of hair, or trichophagia, which subsequently forms trichobezoars in the gastrointestinal tract [2].

In addition to hair loss, the external examination at autopsy may reveal signs of self-harm or other compulsive behaviors that are frequently associated with Rapunzel syndrome. These behaviors can manifest as scars or marks on the skin, often located on accessible parts of the body. Such findings can provide valuable evidence of the individual’s psychological state and potential underlying mental health issues. Detecting these signs helps in constructing a more comprehensive profile of the deceased, which is essential for determining the cause of death and understanding the full scope of the syndrome’s impact on the individual’s life [2].

#### 4.2.8. Histopathological Analysis at Autopsy in Rapunzel Syndrome

The histopathological analysis of tissue samples plays a crucial role in understanding cellular abnormalities associated with Rapunzel syndrome. During an autopsy, microscopic examination of tissue samples can reveal critical insights into the extent of damage caused by trichobezoars. For instance, this examination may detect inflammation or erosion in the gastric and intestinal mucosa, indicative of prolonged irritation from the trichobezoar. Furthermore, the presence of necrotic tissue or ulceration may be observed, suggesting severe damage that might have contributed to the patient’s death.

In addition to examining tissue samples, the analysis of hair structure and composition in trichobezoars is essential for a comprehensive autopsy of Rapunzel syndrome cases. By analyzing the trichobezoar’s structure, forensic experts can assess the size, density, and composition of the mass, which may correlate with the severity of the syndrome. This analysis can also help determine if the trichobezoar was formed over a prolonged period or was a recent development. Identifying secondary complications, such as infection or necrosis, during the autopsy can provide additional context in Rapunzel syndrome cases. The presence of infection might be indicated by abscess formation or signs of systemic inflammatory response. Necrosis, on the other hand, can result from compromised blood supply caused by the pressure exerted by the trichobezoar.

Additionally, biochemical tests can detect any enzymatic imbalances that may have contributed to the tissue’s compromised state [11]. Laboratory tests can reveal deficiencies in essential vitamins and minerals, pointing to long-standing malabsorption issues. Moreover, metabolic panels may indicate imbalances in electrolytes or other metabolic markers, highlighting the physiological stress endured by the body [11].

#### 4.2.9. Post-Mortem Diagnostics of Rapunzel Syndrome

Advanced post-mortem investigations are crucial because the external examination of a body might not reveal any signs of these formations, as demonstrated in cases where large trichobezoars were found in the stomach and small intestine during autopsy [2]. Radiological techniques, such as abdominal CT scans, can provide detailed images that pinpoint the exact location and size of these masses [7]. Magnetic Resonance Imaging (MRI) is another sophisticated tool utilized in post-mortem radiological investigations, particularly for soft tissue analysis in cases of Rapunzel syndrome. MRI’s ability to provide high-resolution images without the use of ionizing radiation makes it an ideal choice for examining delicate soft tissue structures that may be affected by trichobezoars [8]. The technique’s advanced imaging capabilities allow forensic investigators to assess the extent of tissue damage and the presence of any secondary complications, such as inflammation or perforation, that trichobezoars may have caused. While X-rays and CT scans focus more on structural anomalies, MRI provides a complementary perspective by offering detailed insights into the condition of the soft tissues surrounding the trichobezoar. While post-mortem radiological methods have significantly advanced, they have their limitations in investigating cases of Rapunzel syndrome [9], such as the inability to detect certain chemical and physiological changes that occur after death. Moreover, while imaging can provide extensive details about the physical state of the trichobezoar and surrounding tissues, it cannot always offer insights into the functional status of organs at the time of death [9].

#### 4.2.10. Treatment Approaches for Living Subjects

Surgical intervention is often deemed necessary in the treatment of Rapunzel syndrome due to the nature of trichobezoars, which can cause significant gastrointestinal obstruction. The primary surgical approach involves bezoar extraction via laparotomy [13]. This method is often preferred because it allows surgeons to address any potential complications, such as gastric or intestinal perforations [14]. The outcomes of surgical interventions are generally positive, leading to immediate relief of symptoms such as abdominal pain, vomiting, and obstruction [15]. However, the necessity for surgery highlights the importance of early detection and intervention, as delayed treatment can increase the risk of complications and affect recovery outcomes [49,50,51,52,53,54,55,56,57,58,59,60,61,62,63,64,65,66,67,68,69,70,71,72].

Non-surgical treatment options, although less common, play a crucial role in the management of Rapunzel syndrome, particularly in preventing recurrence. These treatments often include psychiatric evaluation and therapy, as trichobezoars are frequently associated with underlying psychiatric conditions such as trichotillomania and trichophagia [16]. Comprehensive psychiatric follow-up is essential to address these behavioral issues, ensuring that the root cause of the syndrome is effectively managed [17].

Cognitive-behavioral therapy (CBT) is particularly effective in helping patients develop healthier coping mechanisms and reduce the compulsive behaviors associated with hair pulling and eating. Psychological evaluation plays a crucial role in understanding the compulsive behavior associated with hair-pulling in individuals with Rapunzel syndrome. This evaluation allows clinicians to identify underlying psychiatric conditions, such as trichotillomania, which is characterized by an irresistible urge to pull out one’s hair [5]. Pharmacological options are also explored as part of the treatment strategy for Rapunzel syndrome, although their effectiveness can vary. Medications such as selective serotonin reuptake inhibitors (SSRIs) may be prescribed to help manage the underlying psychiatric conditions like trichotillomania and trichophagia [3]. While pharmacological treatment can be beneficial, it is typically used in conjunction with psychotherapy to enhance the overall effectiveness of the treatment plan. The integration of medication helps stabilize the patient’s mood and decrease compulsive behaviors, providing a more holistic approach to managing Rapunzel syndrome [73,74,75,76,77,78,79,80,81,82,83,84,85,86,87,88,89,90,91,92].

#### 4.2.11. Prevention and Management Strategies for Rapunzel Syndrome

Educating both patients and their families about the symptoms and potential consequences of hair-pulling behaviors can significantly reduce the risk of developing trichobezoars. Early intervention programs should focus on identifying trichotillomania in its initial stages and incorporating behavioral therapies that aim to alter the patient’s response to stressors. Additionally, incorporating educational sessions in schools and community centers can play a pivotal role in spreading awareness and preventing the syndrome from developing, especially in the pediatric population, who are most at risk [2]. The management of Rapunzel syndrome benefits enormously from a multidisciplinary approach, which combines the expertise of various healthcare professionals. This collaborative treatment strategy typically involves gastroenterologists, psychiatrists, and surgeons working together to address both the physical and psychological aspects of the syndrome [11]. Long-term follow-up and support are integral to the recovery process for individuals affected by Rapunzel syndrome. Psychological support, such as counseling or support groups, can provide encouragement and coping strategies for managing the urges associated with trichotillomania. Additionally, creating a supportive environment at home and school is crucial in reinforcing positive behaviors and reducing stressors that may trigger hair-pulling. Recurrent Rapunzel syndrome (RRS) is exceedingly rare, but when it occurs, it is often due to inadequate follow-up and ongoing psychological issues [17]. To mitigate this risk, a multidisciplinary approach is recommended, involving continued psychological support, regular medical check-ups, and family counseling.

Finally, we propose, for clinical purposes, the following diagnostic protocol [49,50,51,52,53,54,55,56,57,58,59,60,61,62,63,64,65,66,67,68,69,70,71,72,73,74,75,76,77,78,79,80,81,82,83,84,85,86,87,88,89,90,91,92]:

1. Clinical Evaluation:
  - Patient History (look for behavioral signs such as trichotillomania (compulsive hair-pulling); inquire about gastrointestinal symptoms like abdominal pain, nausea, vomiting, and early satiety; document any weight loss or changes in appetite.
  - Physical Examination (assess for signs of abdominal distension or palpable masses; evaluate for possible signs of malnutrition or systemic illness).
2. Laboratory Investigations:
  - Complete Blood Count (CBC) (check for anemia, which is common in cases with gastrointestinal blood loss).
  - Electrolyte Imbalance (monitor for dehydration or electrolyte disturbances due to vomiting or malnutrition).
  - Iron Deficiency (assess for iron deficiency anemia, a frequent complication).
3. Imaging Studies:
  - Ultrasound (first-line imaging to identify gastrointestinal distension or an unusual mass).
  - CT Scan (confirm the presence of bezoars, often showing characteristic gastric distension or signs of small bowel obstruction; helps assess the size and location of the bezoar and any related complications, e.g., perforation).
  - X-ray (may show air-fluid levels indicating gastrointestinal obstruction).
4. Endoscopy:
  - Gastroscopy: (direct visualization of the bezoar within the stomach; helps confirm the diagnosis and assess the extent of the bezoar).
  - Enteroscopy (if necessary): (evaluate for bezoar presence in the small intestine or duodenum, especially in cases with obstruction).
5. Surgical Evaluation:
  - Laparotomy/Laparoscopy (if imaging confirms large bezoar or complications): (invasive exploration may be required if non-invasive methods are inconclusive or if complications like perforation are suspected).
6. Autopsy (Postmortem Investigation in Fatal Cases):
  - Autopsy Investigation (examine the full gastrointestinal tract, including the esophagus, stomach, and intestines, for bezoar remnants and related damage or signs of infections or perforation).
  - Histopathological Analysis (evaluate the presence necrosis or inflammations especially on gastrointestinal tract).

#### 4.2.12. Limitations and Future Perspectives

This review has several limitations that should be acknowledged. First, the analysis relies on case reports and a few retrospective studies, which restricts the ability to generalize findings due to the limited sample size. Furthermore, the lack of longitudinal data makes it difficult to assess long-term outcomes, such as recurrence rates and recovery patterns. A notable skew toward pediatric and female patients is evident, potentially underrepresenting other demographic groups and limiting the broader applicability of the findings.

Looking ahead, there are several important avenues for future research. Large-scale, multicenter epidemiological studies are necessary to better understand the prevalence, risk factors, and demographics of bezoar formation. To improve data comparability, developing standardized guidelines for reporting bezoar-related cases would be invaluable. Exploring the psychological and behavioral factors underlying the condition, such as trichotillomania or pica, could lead to more effective preventive and therapeutic strategies. In terms of treatment, research into innovative, minimally invasive approaches, such as enzymatic therapies or advanced endoscopic techniques, holds significant promise. Preventing recurrence remains a critical area for investigation, with a focus on dietary counseling, behavioral therapy, and long-term follow-ups.

Moreover, future studies should aim to include a more diverse population, encompassing males and elderly patients, to ensure a more inclusive understanding of the condition. Finally, exploring genetic predispositions and biological markers associated with bezoar formation could pave the way for early diagnosis and risk stratification. Addressing these gaps in knowledge will not only enhance clinical management but also improve outcomes for affected individuals.

## 5. Conclusions

Managing Rapunzel syndrome effectively requires a multidisciplinary approach that prioritizes early diagnosis, comprehensive treatment, and long-term follow-up in order to improve patient outcomes. Early recognition of clinical symptoms, such as abdominal pain, nausea, and trichotillomania, along with the use of diagnostic imaging techniques like ultrasound or CT scans, is essential for enabling timely intervention and minimizing complications. Autopsies, particularly in suspected or confirmed cases, play a crucial role in advancing understanding of the syndrome’s long-term effects and rare complications. These investigations, through histopathological, radiological, and forensic analyses, can clarify etiopathogenetic mechanisms, potentially opening new avenues for research in this field.

## Figures and Tables

**Table 1:** Analysis of literature review results.

References	Type of Studies	Age	Sex	Clinical Presentation	Autopsy Finding	Cause of Death	Bezoar Dimensions
[20]	Case report	35	F	Abdominal symptoms	alive	alive	17 × 6 × 5 cm, 6 × 4 cms
[21]	Case report	55	F	Abdominal symptoms, duodenal perforation	alive	alive	unknown
[22]	Case report	16	F	Abdominal symptoms, fatigue, fever	alive	alive	unknown
[14]	Case report	3	F	Pale skin, shortness of breath, fixed and dilated pupils	Stomach was completely filled with a massive trichobezoarfrom the gastroesophageal junction into the proximal jejunum	Aspiration of food due to respiratory distress associated with acute bronchitis	210 g and 25 cm
[23]	Case report	18	F	Abdominal symptoms, anemia, dehydration	alive	alive	75 cm
[24]	Case report	18	F	Abdominal symptoms	alive	alive	120 cm
[17]	Case report	25	F	Abdominal bloating, early satiety, anemia	alive	alive	unknown
[25]	Case report	6	F	Abdominal symptoms	alive	alive	100 g, 11 cm × 16 cm
[26]	Case report	25	F	Abdominal symptoms, gastrointestinal mass	alive	alive	unknown
[15]	Case report	16	F	Abdominal symptoms, anorexia	alive	alive	165 × 90 × 85 mm
[27]	Case report	17	F	Loss of weight, abdominal symptoms	alive	alive	480 × 70 × 50 mm
[4]	Case report	14	F	Loss of weight, anemia, anorexia, abdominal symptoms	alive	alive	unknown
[28]	Case report	16	F	Prostration, anorexia, loss of weight, abdominal symptoms, anemia, constipation	alive	alive	22 × 10 cm
[29]	Case report	7	F	Abdominal symptoms	alive	alive	unknown
[8]	Case report	10	F	Iron deficiency anemia, alopecia areata, flu-like symptoms, abdominal symptoms, anorexia, fatigue	Three trichobezoars, occupying the stomach, jejunum, and ileum	Peritonitis secondary to small bowel perforations	unknown
[2]	Case report	4	F	Abdominal symptoms	Three large trichobezoars localized in the stomach and the small intestine	Collateral bronchoaspiration of dietary material	unknown
[19]	Retrospective study	10 patients	F	Abdominal symptoms, complications (e.g., small bowel obstruction, severe gastric dilation)	alive	alive	unknown

## Data Availability

Not applicable to this article as no datasets were generated.
